# Mild decrease in *TBX20* promoter activity is a potentially protective factor against congenital heart defects in the Han Chinese population

**DOI:** 10.1038/srep23662

**Published:** 2016-04-01

**Authors:** Li-Wei Yu, Feng Wang, Xue-Yan Yang, Shu-Na Sun, Yu-Fang Zheng, Bin-Bin Li, Yong-Hao Gui, Hong-Yan Wang

**Affiliations:** 1 Children’s Hospital, Institute of Reproduction and Development. Collaborative Innovation Center of Genetics and Development, Fudan University, Shanghai, China; 2 The State Key Laboratory of Genetic Engineering. MOE Key Laboratory of Contemporary Anthropology, Collaborative Innovation Center of Genetics and Development, School of Life Science, Fudan University, Shanghai, China

## Abstract

Congenital heart defects (CHDs) are one of the most common human birth defects worldwide. TBX20 is a crucial transcription factor for the development of embryonic cardiovascular system. Previous studies have demonstrated that mutations in the *TBX20* coding region contribute to familial and sporadic CHD occurrence. However, it remains largely unknown whether variants in the *TBX20* regulatory region are also related to CHDs. In this study, we sequenced the 2 kb region upstream of the *TBX20* transcription start site in 228 CHD patients and 292 controls in a Han Chinese population. Among the 8 single nucleotide polymorphisms (SNPs) identified, six SNPs are in strong linkage disequilibrium and the minor alleles are associated with lower CHD risk (for rs10235849 chosen as tag SNP, *p* = 0.0069, OR (95% CI) = 0.68 (0.51–0.90)). Functional analysis showed that the minor alleles have lower transcriptional activity than major alleles in both human heart tissues and three cell lines. The electrophoretic mobility shift assay suggested that *TBX20* minor alleles may exhibit higher binding affinity with certain transcription repressors. Our results indicate that a moderately lower TBX20 activity potentially reduces CHD risk in the Han Chinese population, providing new insight in the study of CHD etiology.

Congenital heart defects (CHDs) are among the most common human structural birth defects, and a leading cause of infant death. The worldwide incidence of CHDs is approximately 8% in newborns^1^. Most patients require surgery, and some infants develop functional cardiac impairments post-surgery that compromises their entire lives. Current studies have found that both environmental and genetic factors contribute to the occurrence of CHDs^2^,^3^,^4^,^5^. The complicated network of transcription factors plays a crucial role during cardiogenesis^6^. These factors interconnect upstream signaling molecules with downstream target genes, which influences cardiac cell migration, proliferation and differentiation^7^.

The function of transcription factors can be affected by either conformational changes or dosage. Dosage of transcription factors could be affected by genetic variants in their regulatory regions, which have been reported to associate with CHD risk^8^,^9^. One SNP identified in *TBX1* promoter enhances TBX1 expression level and is associated with increased risk of ventricular septal defect (VSD)^10^. Another SNP in *Wnt5a* 5’untranslated regions (UTR) decreases *Wnt5a* expression level and is associated with reduced risk of cardiac conotruncal malformations^11^. Furthermore, SNPs in *GATA4* 3’UTR were also reported to increase risk of CHDs by microRNA regulation^12^. In addition to those studies on individual genes, genome-wide association studies also suggest that a large number of non-coding variants contribute to the risk of cardiovascular disease^13^,^14^. These results demonstrated that genetic variants in regulatory regions significantly influence gene expression and cardiogenesis. Thus, we hypothesized that functional regulatory variations in vital transcription factor genes could modulate gene expression and CHD susceptibility.

TBX20 is an important transcription factor for cardiac development. Tbx20 null mice stop development at E9.0 and die around E10.5, and display outflow tract defects with underdeveloped short heart tubes, a generalized developmental arrest, and lack of chamber differentiation^15^,^16^,^17^. Tbx20 also actively interacts with other vital transcription factors and interacts with the Wnt and Bmp signaling pathways^8^,^18^. Indeed *TBX20* expression in humans is primarily located in the first heart field (FHF) and the second heart field (SHF) throughout embryonic cardiogenesis, and is involved in valve elongation and remodeling^18^,^19^.

Tbx20 also has a dosage effect. Takeuchi found that a 95% reduction of Tbx20 results in embryos lacking an outflow tract, whereas mice with a 60% reduction of Tbx20 have less severe heart developmental defects, presenting with impaired outflow tract septation, right ventricular hypoplasia, and defective valve formation^16^. It’s worth noting that heterozygous *Tbx20* knockout mice are viable and do not show any detectable heart defects^20^,^21^.

However, the effect of TBX20 dosage in human CHDs has not been fully investigated, and genetic variants in the promoter region have rarely been reported. To investigate the effect of *TBX20* expression level on CHD risk, we sequenced the *TBX20* promoter region in CHD patients/controls. The minor allele of rs10235849 and the haplotype ATC are associated with reduced CHD risk and contribute to attenuated *TBX20* transcription in functional analyses. We demonstrated that a mild decrease in *TBX20* expression is related to a lower risk of CHD occurrence in a Han Chinese population.

## Results

### P6-Minor significantly reduces susceptibility of CHDs in a Han Chinese population

**Figure 1:**
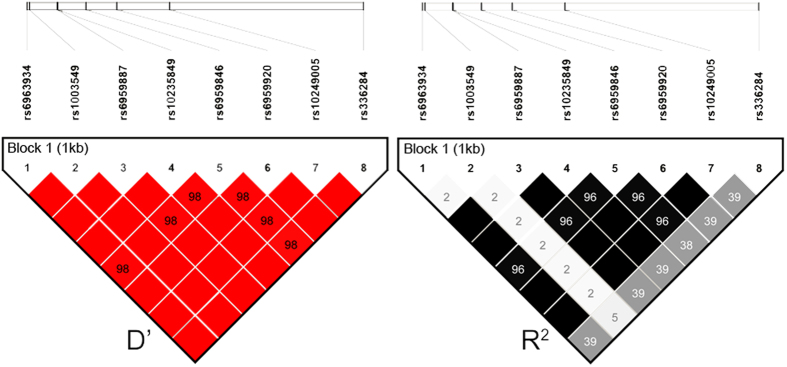
Linkage disequilibrium analysis of eight associated SNPs present in *TBX20* promoter region in our samples. Six SNPs are in strong LD. Each box represents the LD relationship between two SNPs. The white line on the top shows the relative physical positions of the SNPs on the chromosome. rs6963934, rs6959887, rs10235849, rs6959846, rs6959920 and rs10249005 are in strong LD (D’ > 0.9 and R^2^ > 0.8).

We identified eight genetic variants with minor allele frequency (MAF) greater than 10% in approximate 2 kb of the *TBX20* promoter region in 228 CHD patients and 192 healthy controls. All samples are from Anhui province, in southern China. We also included 100 CHS (China South) samples from the 1000 Genomes Project (http://browser.1000genomes.org/index.html) as controls. Our association study was ultimately based on 228 cases and 292 controls. Among these eight SNPs, six SNPs (rs6963934, rs6959887, rs10235849, rs6959846, rs6959920 and rs10249005, termed as P6) are in strong LD (Fig. 1). Therefore, the genotypes of the six SNPs change together and these SNPs only form 2 haplotypes, P6-Major/P6-Minor (Table S2). The association study suggested that P6-Minor significantly reduces CHD risk in Han Chinese (*p* = 0.0069, OR = 0.68, 95%CI = 0.51–0.90, Data from tag SNP rs10235849) (Table 1). The power of rs10235849 is 0.48, calculated by R with package “pwr”. The other two SNPs (rs1003549 and rs336284) do not show LD with the six SNPs mentioned above. Further association studies based on the remaining two SNPs showed that the minor allele of rs336284 is associated with a reduced CHD risk (*p* = 0.01, OR = 0.72, 95%CI = 0.56–0.93), while rs1003549 is not (*p* = 0.54, OR = 0.85, 95%CI = 0.50–1.44).

To analyze the complete correlative extent of all eight SNPs, we took rs10235849 (tag SNP), rs336284 and rs1003549 together to reconstruct haplotypes of all the SNPs using Haploview version 4.2. We found four haplotypes with frequencies greater than 5% in our samples (Table 2). The association analysis between haplotype AAT, ATC, AAC, GAT and CHD risk showed that haplotype ATC significantly decreases the risk of CHDs, compared with haplotype AAT, which consisted of all of the major alleles (*p* = 0.0031, OR = 0.61, 95%CI = 0.44–0.85).

### P6-Minor decreases *TBX20* promoter activity

**Figure 2:**
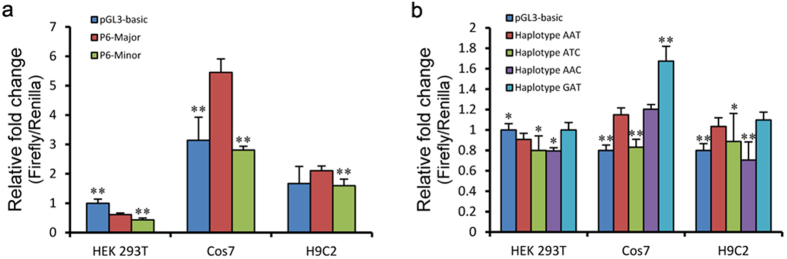
Luciferase assay to analyze transcriptional activity. (**a**) Promoter activity analysis using dual-reporter luciferase system in three cell lines. P6-Major contains all major alleles of six positive SNPs, and P6-Minor contains all six minor alleles. Luciferase assay showed that P6-Minor has decreased transcriptional activity compared with major alleles in three different cell lines. (n > 3, *p < 0.05, **p < 0.01, compared with P6-Major); (**b**) Promoter activity analysis of four haplotypes using dual-luciferase reporter system in three cell lines (n > 3, *p < 0.05, **p < 0.01, compared with Haplotype AAT in the same cell line).

Given that all of the SNPs are located in the promoter region of *TBX20*, we speculated that they might interfere with promoter activity. We tested the promoter activity of P6-Major and P6-Minor in a dual-luciferase reporter system. Human embryo kidney epithelial cell line (HEK 293T), monkey kidney fibroblast-like cell line (Cos7), and rat heart tissue cell line (H9C2) were used in our cellular studies. We observed that the P6-Minor shows lower transcriptional activity by 28.8%, 48.4% and 24.1% respectively, compared with the activity of P6-Major in three cell lines, respectively (Fig. 2a). As the minor alleles are found to be protective (OR = 0.68), this result indicated that a lower expression level of *TBX20* may decrease CHD risk.

**Figure 3:**
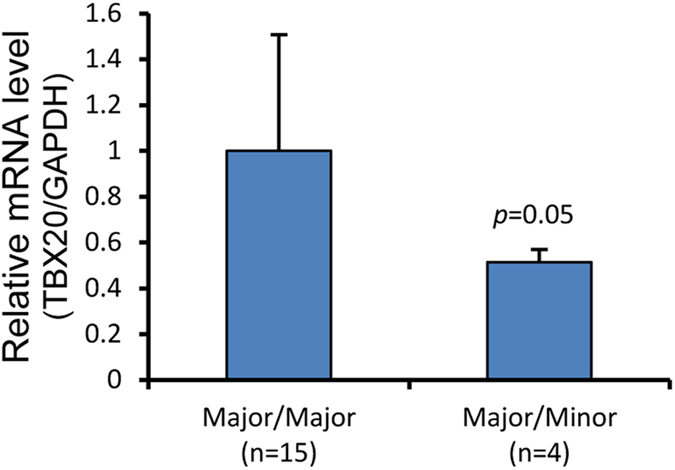
Analysis of *TBX20* mRNA level in human tissue samples. After genotyping, *TBX20* mRNA level was quantified by qRT-PCR, using *GAPDH* as internal control. Fifteen samples were Major/Major, four samples were Major/Minor. Relative mRNA level of two genotypes were calculated by 2^−Δct^. Major/Minor tends to have a lower mRNA level than Major/Major (n = 19, *p* = 0.05).

To further evaluate the transcriptional level of *TBX20*, qRT-PCR was used to evaluate mRNA levels in human tissue samples after genotyping (Table 3). Carriers with the P6-Minor haplotype have a significantly lower mRNA level (Fig. 3). This indicated that P6-Minor attenuates *TBX20* promoter activity at the transcriptional level.

In addition, the promoter involving haplotype ATC has a lower transcriptional activity than haplotype AAT by 12%, 27.7% and 14% in HEK 293T, Cos7 and H9C2, respectively (Fig. 2b). Meanwhile, haplotype ATC is also associated with a lower risk of CHDs according to the association analysis. This indicated that minor alleles of rs336284 also contribute to a lower promoter activity to some extent.

### Minor alleles of rs10235849 and rs6959887 have higher binding affinity with specific nuclear proteins

**Figure 4:**
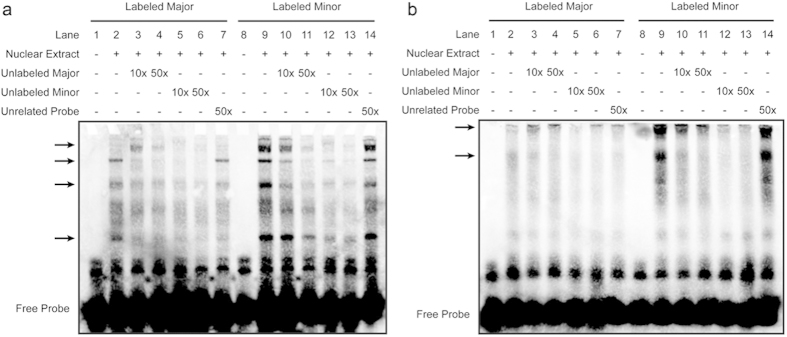
Analysis of DNA binding affinity with two genotypes. (**a**) Binding affinity of Major/Minor-probes with HEK 293T nuclear proteins. Four arrows highlight four specific nuclear proteins that interact with Major/Minor-probes. All four proteins have higher binding affinity with Minor-probe. (**b**) Binding affinity of Major/Minor-probes with H9C2 nuclear proteins. Two arrows highlight two specific nuclear proteins that interact with Major/Minor-probes. Both proteins have higher binding affinity with the Minor probe.

As we found that P6-Minor decreases *TBX20* promoter activity in three cell lines, we questioned whether these changes might due to different binding affinity to some transcription factors. Using available bioinformatic tools (Alibaba, Genomatix and TFsearch), we produced a Major-probe and a Minor probe, including the site of rs10235849 and rs6959887. EMSA was performed to clarify the different transcription factor binding affinity due to these genetic variants. Four and two specific shifted bands were obtained using HEK 293T and H9C2 nuclear extracts, respectively (Fig. 4). We noticed that in both cases, Minor-probe has a higher binding affinity with some nuclear proteins (compare lane 9 with lane 2). Competition assays using unlabeled probes also demonstrated a higher binding affinity of transcription factors with minor alleles. As P6-Minor shows decreased *TBX20* promoter activity compared with P6-Major, we presumed that those specific binding proteins are most likely transcription repressors, the details of which deserve further investigation.

## Discussion

We identified rs10235849 (tag SNP) as being associated with a modest decrease in *TBX20* expression and reduced risk of CHDs in a south Chinese population. Septal defects account for 91.3% of our case samples, with VSD as major CHD subtype (68.8%), and atrial septal defects (ASDs) as the second most common CHD subtype (22.5%). Since TBX20 is one of the most important transcription factors expressed in both FHF and SHF^22^, TBX20 dosage is a critical factor associated with CHD risk.

Besides our study, there were two other studies that focused on genetic variants of *TBX20*. One study included 25 CHD patients in their association study, and found two SNPs associated with CHD risk in the *TBX20* coding region^23^. Another association study identified three SNPs in the 1,200 bp upstream of *TBX20* TSS in 265 VSD patients *vs* 242 controls^24^. However, none of them were related to CHD risk. Notably, their study was conducted in a northern Chinese population rather than a southern Chinese population like the one in our study^24^. Different genetic background should be considered when the results are not consistent. Further confirmatory studies in large cohorts are needed.

We significantly extended the sequencing region of *TBX20* promoter regulatory region in our study. This extension led to the discovery of the P6 region. The P6-Minor haplotype was associated with reduced risk of CHDs and a downregulation of TBX20 as a protection effect. This confirmed our hypothesis that decreased TBX20 is associated with reduced CHD risk. Tbx20 is one of the earliest expressed transcription factors and it acts as a repressor in heart development and regulates other transcription factors^21^. For example, Tbx2 and Tbx5 are two regulating targets of Tbx20 and both of them are indispensable in heart development^25^,^26^,^27^. In mice, Tbx20 restricts Tbx2 expression to the atrioventricular canal region, the process of which is essential to proper chamber septation^8^. In Chakraborty’s study, Nkx2.5 Cre-mediated *Tbx20* overexpression mice resulted in smaller hearts, thin ventricles, and decreased trabeculation^28^. They also confirmed the inhibitory effect on Tbx2 and Tbx5 expression, as well on the ERK1/2 MAPK signaling pathway which is morphogenetically critical in chamber formation^28^. In humans, evidence has accumulated that decreased expression of TBX5 and TBX2 contributes to CHDs^29^,^30^. Therefore, a possible mechanism of action is that lower TBX20 level could allow upregulation of its target genes, including TBX2, TBX5, etc. This may explain how a moderately decreased *TBX20* expression level is linked with a lower risk of CHDs.

We observed that several transcription factors bind to the *TBX20* promoter region in our EMSA results. Unfortunately, we were unable to determine the identity of these transcription factors. Three potential transcription factors: CDX1, POU4f3 or POU1f1, were predicted by three different bioinformatic tools. We subsequently co-transfected each transcription factor with P6-Major/P6-Minor in HEK 293T and Cos7 cell lines. We failed to observe any repressive effect on TBX20 by these three transcription factors. Therefore, we think the repression effect may occur either through unidentified transcription factors, or a transcription factor complex. Our future goals involve attempts to resolve this issue.

To summarize, we identified minor alleles of a set of SNPs that are enriched in the normal population compared with CHD patients. Subsequently, functional studies in three cell lines showed that minor alleles have lower transcriptional activity and a higher binding affinity with certain nuclear proteins. Our research provides more information on the potential role of the regulatory region in terms of transcriptional activity and illuminates the molecular mechanism of CHD pathogenesis.

## Materials and Methods

### Study subjects

All CHD patients (n = 228, 18.6 ± 12.1 years) were recruited from Second Hospital of Anhui Medical University (Hefei, Anhui province, China). Routine clinical diagnoses were conducted, and patients with CHDs were included. The majority of our case samples are septal defects, including 68.8% with VSD and 22.5% with ASD. The age at diagnosis ranged from early childhood to adult. All unrelated healthy controls (n = 192, 17.9 ± 1.3 years) were collected from new recruits in Anhui province of China. One hundred individuals from the China South population in the 1000 Genomes project were also added to the controls additionally. All subjects are genetically ethnic Han Chinese. Tissue samples were obtained from children who underwent cardiac surgery in the Children’s Hospital of Fudan University, Shanghai.

The present studies on our samples were conducted in accordance with the Declaration of Helsinki. Protocols used in this work were reviewed and approved by the Ethics Committee of the School of Life Sciences, Fudan University prior to the commencement of the study. Written consent was obtained from the parents and/or guardians of the children.

### Single nucleotide polymorphisms (SNPs) identification and genotyping

Genomic DNA was prepared from peripheral leukocytes using a DNA extraction kit (LifeFeng, China), and quantified using a NanoDrop2000 (Thermo Fisher Scientific, USA). Since both isoforms of *TBX20* lack 3’UTR, a promoter region approximately 2 kb upstream of TSS was amplified by PCR and sequenced using BigDye Terminator v3.0 reagents on a 3730 DNA Analyzer (Applied Biosystems, USA) in all individuals. The PCR primers were designed according to the genomic sequence of the human *TBX20* gene (NC_000007.14; 35202430–35254100) and are listed in Table S1. Genotyping was analyzed using the SeqMan software (DNASTAR, USA).

### Quantitative real-time PCR

Total RNA was extracted from the human heart tissue samples using a total RNA kit (DP419, Tiangen Biotech, Beijing), converted to cDNA using Fast Quant RT kit (KR-106, Tiangen Biotech, Beijing), and underwent qPCR reaction with SuperReal PreMix Plus(FP205, Tiangen Biotech, Beijing). The *TBX20* mRNA level was determined by quantitative real-time RT-PCR using the ABI Prism 7900 sequence detection system with *GAPDH* as an internal control. Each reaction was performed in triplicate. The primers were designed to be able to test both isoforms of *TBX20* mRNA (Table S1).

### Plasmid constructs, cell culture, and luciferase assays

To construct luciferase reporter plasmids with the *TBX20* promoter, we amplified 955-bp fragments from −2,373 to −1,419 of both major alleles/minor alleles containing 6 SNPs in strong LD (rs6963934, rs6959887, rs10235849, rs6959846, rs6959920 and rs10249005) and subcloned them into the KpnI/XhoI sites of the pGL3-basic vector (Promega, USA) (Table S1), which was named P6-Major/P6-Minor.

To study the function of each haplotype, we amplified longer fragments containing 1,743 bp from −1,933 to −191, involving all 8 SNPs, and subcloned them into NheI/XhoI sites of the pGL3-basic vector (Table S1). We then performed site-directed mutations in accordance with all four haplotypes (Tables 2, S1) and named them Haplotype AAT, Haplotype ATC, Haplotype AAC and Haplotype GAT.

Cos7 (1 × 10^5^), HEK 293T (1.2 × 10^5^) and H9C2 (2 × 10^5^) were seeded in 24-well cell culture plates 24 hrs before the transient transfections. All cell lines were transfected with 300 ng/well P6-Major/P6-Minor, Haplotype AAT/Haplotype ATC/Haplotype AAC/Haplotype GAT, and 10 ng/well pRL-TK plasmid (Promega, USA) as an internal control, using Lipofectamine 2000 (Life Technologies, USA). Samples were assayed for luciferase activity using the dual-luciferase reporter assay system (Promega, USA) 24 hrs post-transfection according to the manufacturer’s instructions.

### Probe design and EMSA

We used three online bioinformatic algorithms to predict the effect of genetic variants in *TBX20* promoter: Alibaba (http://www.gene-regulation.com/pub/programs/alibaba2/index.com), Genomatix (https://www.genomatix.de/?s=e12a7dd6f4db03e3a3 e072ea9b13e806) and TFsearch (www.cbrc.jp/research/db/TFSEARCH.html). All three tools indicated a change of nuclear protein binding at the sites of rs10235849 and rs6959887. Therefore, EMSA probes including the two SNPs were designed from −1,666 to −1,639 in two genotypes. Since rs10235849 and rs6959887 were in complete LD, the Major-probe was consisted of major alleles of rs10235849 and rs6959887, and Minor-probe was consisted of all minor alleles (Table S1). In addition, rs10235849 was also taken as tag SNP in our study because it was included in EMSA probes.

Nuclear extracts were prepared from HEK 293T cells and H9C2 cells using the NE-PER Nuclear and Cytoplasmic Extraction kit (Life Technologies, USA) and stored at −80 °C before use. Protein concentration was measured using a NanoDrop2000 (Thermo Fisher Scientific, USA). The probes were incubated with nuclear proteins and ran on a 6% polyacrylamide gel. All the procedures were followed as described in the EMSA kit instructions (Life Technologies, USA).

### Statistical analysis

Hardy–Weinberg equilibrium was evaluated in the controls and in the association study for both the CHD cases and the controls were compared using the χ^2^ test. We calculated the odds ratios (ORs) and 95% confidence intervals (CIs). The association study between tag SNP and CHD risk were performed using the SNPStats (http://bioinfo.iconcologia.net/snpstats/start.htm), as well as the association analyses between Haplotype AAT, ATC, AAC, GAT and CHD risk. We reconstructed haplotypes of the SNPs using Haploview version 4.2.

Differences in cellular study were evaluated using the one-way ANOVA. The statistical difference between Major/Major and Major/Minor genotypes of *TBX20* expression levels in human tissue samples was analyzed by independent samples T test. The one-way ANOVA and independent samples T test were two-tailed with *p* < 0.05 as the significance level and were performed using the SPSS 16.0 software (SPSS, USA).

## Additional Information

**How to cite this article**: Yu, L.-W. *et al.* Mild decrease in *TBX20* promoter activity is a potentially protective factor against congenital heart defects in the Han Chinese population. *Sci. Rep.* **6**, 23662; doi: 10.1038/srep23662 (2016).

## Supplementary Material

Supplementary Information

## Figures and Tables

**Table 1:** Association of all polymorphisms in *TBX20* promoter region with CHDs in case-control studies.

db SNP No.	Genotype	Pattern	Case	Control	P value	OR (95% CI)	Hardy-Weinberg equilibrium*
rs1003549	Codominant	AA/AG/GG	201/23/0	258/29/3			
	Dominant	AA/AG + GG	201/23	258/32			
	Recessive	AA + AG/GG	224/0	287/3			
	Overdominant	AA + GG/AG	201/23	261/29			
	Log additive	A/G	–	–	0.54	0.85 (0.50–1.44)	0.074
rs10235849 tag SNP	Codominant	AA/AT/TT	138/78/10	148/115/27			
	Dominant	AA/AT + TT	138/88	148/142			
	Recessive	AA + AT/TT	216/10	263/27			
	Overdominant	AA + TT/AT	148/78	175/115			
	Log additive	A/T	–	–	**0.0069**	**0.68 (0.51–0.90)**	**0.57**
rs336284	Codominant	TT/TC/CC	73/113/37	76/139/76			
	Dominant	TT/TC + CC	73/150	76/215			
	Recessive	TT + TC/CC	186/37	215/76			
	Overdominant	TT + CC/TC	110/113	152/139			
	Log additive	T/C	–	–	0.01	0.72 (0.56–0.93)	0.48

All *p*-values were calculated based on χ^2^. OR, odds ratio; CI, confidence interval.

^*^Hardy-Weinberg equilibrium of control population.

**Table 2:** Haplotype frequency estimation and haplotype association with response (n = 520): Haplotype AAT, ATC, AAC, GAT are four haplotypes with a frequency over 5% in our samples.

	rs1003549	rs10235849(tag SNP)	rs336284	Freq.	Control	Case	Cumulativefrequency	OR (95%CI)	P-value
Haplotype AAT	A	A	T	0.473	0.4442	0.4393	0.5251	1	—
Haplotype ATC	A	T	C	0.2583	0.2958	0.2904	0.2095	**0.61 (0.44**–**0.85)**	**0.0031**
Haplotype AAC	A	A	C	0.2126	0.1945	0.2102	0.2155	0.86 (0.62–1.21)	0.4
Haplotype GAT	G	A	T	0.0561	0.0654	0.0601	0.0491	0.71 (0.40–1.26)	0.24

All *p*-values were calculated based on χ^2^. OR, odds ratio; CI, confidence interval.

Tag SNP is involved in statistical calculation on behalf of other five SNPs in strong LD as listed in Table S2.

**Table 3:** Genotypes of human tissue samples.

	Genotypes of tissue samples		
	Major/Major	Major/Minor	Minor/Minor
rs10235849*A > T	A/A	A/T	T/T
Case number in mRNA analyses	15	4	0

^*^Indicates tag SNP.
