# Supplementary material for: Mild decrease in TBX20 promoter activity is a potentially protective factor against congenital heart defects in the Han Chinese population
Source: Sci Rep. 2016 Apr 1;6:23662. doi: 10.1038/srep23662 (PMC4817057; doi:10.1038/srep23662)
Supplement: Supplementary Information [file srep23662-s1.doc]

**Mild decrease in *TBX20* promoter activity is a potentially protective factor against congenital heart defects in the Han Chinese population**

Li-Wei Yu^1#^, Feng Wang^1#^, Xue-Yan Yang^2^, Shu-Na Sun^1^, Yu-Fang Zheng^2^, Bin-Bin Li^2^, Yong-Hao Gui^1^*, and Hong-Yan Wang^2^*

**Table S1. Primers and probes used in the study**

|  | Sequence (5` to 3`) |
| --- | --- |
| P6-Major/Minor cloning F | AACCATAACATTCCCCGACCT |
| P6-Major/Minor cloning R | GGGTAATCCGCTTCGCTTG |
| Haplotypes cloning F | TTTGCCACACCCTGTTGAAG |
| Haplotypes cloning R | GCACAGGTTCAGGGAAAAGG |
| Point mutation (rs1003549) F | GCCTTCCACTCGATACAAAGAAATG |
| Point mutation (rs1003549) R | CACGGAAGGTGAGCTATGTTTCTTT |
| Point mutation(rs336284) F | CCGCCCGGGGCTGCGAGACCAGGTG |
| Point mutation(rs336284) R | CGGGCCCCGACGCTCTGGTCCACCC |
| *TBX20* Genotyping F | AAGGGGTCTGGATTGGGTCT |
| *TBX20* Genotyping R | CAAGGTGCTGGATGAAGGTG |
| qRT-PCR F | GAGCTTTGGGACAAATTCCA |
| qRT-PCR R | TACTTGGCCTCAGGATCCAC |
| Major Probe for EMSA F | Biotin-CAGCAAAAAATTTAAGAATAGAAACGGA |
| Major Probe for EMSA R | Biotin-TCCGTTTCTATTCTTAAATTTTTTGCTG |
| Minor Probe for EMSA F | Biotin-CAGCAAAAAATTCATGAATAGAAACGGA |
| Minor Probe for EMSA R | Biotin-TCCGTTTCTATTCATGAATTTTTTGCTG |

**Table S2.** **Haplotypes of six highly-linked SNPs in the study**

P6-Major is composed of all the major alleles of SNPs, and P6-Minor is composed of all the minor alleles of the SNPs.

| **Haplotype** | rs6963934  T>C | rs6959887  T>C | rs10235849*  A>T | rs6959846  G>C | rs6959920 C>A | rs10249005 T>C |
| --- | --- | --- | --- | --- | --- | --- |
| **P6-Major** | T | T | A | G | C | T |
| **P6-Minor** | C | C | T | C | A | C |

* indicates tag SNP
